# Loss to follow-up of HIV-infected women after delivery: The Swiss HIV Cohort Study and the Swiss Mother and Child HIV Cohort Study

**DOI:** 10.7448/IAS.17.4.19535

**Published:** 2014-11-02

**Authors:** Karoline Aebi-Popp, Roger Kouyos, Barbara Bertisch, Cornelia Staehelin, Irene Hoesli, Martin Rickenbach, Claire Thorne, Claudia Grawe, Enos Bernasconi, Matthias Cavassini, Begona Martinez de Tejada, Marcel Stoeckle, Thanh Lecompte, Christoph Rudin, Jan Fehr

**Affiliations:** 1 Department of Infectious Diseases, University Hospital Bern, Bern, Switzerland; 2 Department of Infectious Diseases, University Hospital Zürich, Zürich, Switzerland; 3 Department of Infectious Diseases, Cantonal Hospital St. Gallen, St. Gallen, Switzerland; 4 Department of Obstetrics, University Hospital Basel, Basel, Switzerland; 5 Data Centre of the Swiss HIV Cohort Study, Institute for Social and Preventive Medicine, Lausanne, Switzerland; 6 UCL Institute of Child Health, University College London, MRC Centre of Epidemiology for Child Health, London, UK; 7 Department of Obstetrics, University Hospital Zürich, Zürich, Switzerland; 8 Department of Infectious Diseases, Cantonal Hospital Lugano, Lugano, Switzerland; 9 Department of Infectious Diseases, University Hospital Lausanne, Lausanne, Switzerland; 10 Department of Obstetrics, University Hospital Geneva, Geneva, Switzerland; 11 Department of Infectious Diseases, University Hospital Basel, Basel, Switzerland; 12 Department of Infectious Diseases, University Hospital Geneva, Geneva, Switzerland; 13 Department of Nephrology, University Children's Hospital, Basel, Switzerland

## Abstract

**Introduction:**

HIV-infected pregnant women are very likely to engage in HIV medical care to prevent transmission of HIV to their newborn. After delivery, however, childcare and competing commitments might lead to disengagement from HIV care. The aim of this study was to quantify loss to follow-up (LTFU) from HIV care after delivery and to identify risk factors for LTFU.

**Methods:**

We used data on 719 pregnancies within the Swiss HIV Cohort Study from 1996 to 2012 and with information on follow-up visits available. Two LTFU events were defined: no clinical visit for >180 days and no visit for >360 days in the year after delivery. Logistic regression analysis was used to identify risk factors for a LTFU event after delivery.

**Results:**

Median maternal age at delivery was 32 years (IQR 28–36), 357 (49%) women were black, 280 (39%) white, 56 (8%) Asian and 4% other ethnicities. One hundred and seven (15%) women reported any history of IDU. The majority (524, 73%) of women received their HIV diagnosis before pregnancy, most of those (413, 79%) had lived with diagnosed HIV longer than three years and two-thirds (342, 65%) were already on antiretroviral therapy (ART) at time of conception. Of the 181 women diagnosed during pregnancy by a screening test, 80 (44%) were diagnosed in the first trimester, 67 (37%) in the second and 34 (19%) in the third trimester. Of 357 (69%) women who had been seen in HIV medical care during three months before conception, 93% achieved an undetectable HIV viral load (VL) at delivery. Of 62 (12%) women with the last medical visit more than six months before conception, only 72% achieved an undetectable VL (p=0.001). Overall, 247 (34%) women were LTFU over 180 days in the year after delivery and 86 (12%) women were LTFU over 360 days with 43 (50%) of those women returning. Being LTFU for 180 days was significantly associated with history of intravenous drug use (aOR 1.73, 95% CI 1.09–2.77, p=0.021) and not achieving an undetectable VL at delivery (aOR 1.79, 95% CI 1.03–3.11, p=0.040) after adjusting for maternal age, ethnicity, time of HIV diagnosis and being on ART at conception.

**Conclusions:**

Women with a history of IDU and women with a detectable VL at delivery were more likely to be LTFU after delivery. This is of concern regarding their own health, as well as risk for sexual partners and subsequent pregnancies. Further strategies should be developed to enhance retention in medical care beyond pregnancy.

